# 1314. Descriptive Epidemiology of 30-day Readmissions among Survivors of Hospitalization with Bacterial Nosocomial Pneumonia in the US, 2012-2019

**DOI:** 10.1093/ofid/ofab466.1506

**Published:** 2021-12-04

**Authors:** Marya Zilberberg, Brian Nathanson, Laura A Puzniak, Noah Zilberberg, Andrew F Shorr

**Affiliations:** 1 EviMed Research Group, LLC, Goshen, MA; 2 OptiStatim, LLC, Longmeadow, MA; 3 Merck & Co., Inc., Kenilworth, New Jersey; 4 EviMed Research Group, LLC & University of Massachusetts, Amherst, Amherst, Massachusetts; 5 Medstar Washington Hospital Center, Washington, DC

## Abstract

**Background:**

Nosocomial pneumonia (NP) remains associated with excess morbidity and mortality. The effect of NP on other measures of outcome and quality, such as re-admission at 30 days, remains unclear. Moreover, differing types of NP may have varying impacts on re-admissions.

**Methods:**

We conducted a multicenter retrospective cohort study within the Premier Research database, a source containing administrative, pharmacy, and microbiology data. The rate of rehospitalization at 30 days following the index discharge served as our primary endpoint. We compared NP patients readmitted with pneumonia (RaP) as the principal diagnosis to those readmitted for other reasons (RaO). We also compared readmission rates as function of the type of NP: ventilator-associated bacterial pneumonia (VABP), ventilated hospital-acquired bacterial pneumonia (vHABP), and non-ventilated HABP (nvHABP).

**Results:**

Among 17,819 patients with NP, 14,123 (79.3%) survived to discharge, of whom 2,151 (15.2%) required an acute readmission within 30 days of index discharge. Of these, 106 (4.9%) were RaP, and the remainder were RaO. At index hospitalization, RaP patients were older (mean age (SD) 67.4 (13.9] vs. 63.0 (15.2) years), more likely medical (44.3% vs. 36.7%), and less chronically ill (median [IQR] Charlson scores (3 [2-5] vs. 4 [2-5]) than persons with RaO. Bacteremia (10.4% vs. 17.5%), need for vasopressors (15.1% vs. 20.0%), dialysis (9.4% vs. 16.5%), and/or sepsis (9.4% vs. 16.5%) or septic shock 14.2% vs. 17.1%) occurred less frequently in the RaP group. With respect to NP type, nvHABP was most common in RaP (47.2%) and VABP in RaO (38.1%).

**Conclusion:**

One in seven survivors of a hospitalization complicated by NP requires an acute rehospitalization within 30 days. However, few of these readmissions had a principal diagnosis of pneumonia, irrespective of NP type. This suggests that short-term readmission does not capture the quality of care initially delivered to patients for their NP. Of the 5% of NP subjects with RaP, the plurality initially suffered from nvHABP.

**Disclosures:**

**Marya Zilberberg, MD, MPH**, **Cleveland Clinic** (Consultant)**J&J** (Shareholder)**Lungpacer** (Consultant, Grant/Research Support)**Merck** (Grant/Research Support)**scPharma** (Consultant)**Sedana** (Consultant, Grant/Research Support)**Spero** (Grant/Research Support) **Brian Nathanson, PhD**, **Lungpacer** (Grant/Research Support)**Merck** (Grant/Research Support)**Spero** (Grant/Research Support) **Laura A. Puzniak, PhD**, **Merck & Co., Inc.** (Employee) **Andrew F. Shorr, MD, MPH, MBA**, **Merck** (Consultant)

